# The preclinical and clinical progress of cell sheet engineering in regenerative medicine

**DOI:** 10.1186/s13287-023-03340-5

**Published:** 2023-04-27

**Authors:** Danping Hu, Xinyu Li, Jie Li, Pei Tong, Zhe Li, Ge Lin, Yi Sun, Juan Wang

**Affiliations:** 1 Institute of Reproductive and Stem Cell Engineering, School of Basic Medical Science, Central South University, Changsha, 410008 China; 2 Shanghai Biomass Pharmaceutical Product Evaluation Professional Public Service Platform, Center for Pharmacological Evaluation and Research, China State Institute of Pharmaceutical Industry, Shanghai, 200437 China; 3 HANGZHOU CHEXMED TECHNOLOGY CO., LTD, Hangzhou, 310000 China; 4 Hospital of Hunan Guangxiu, Medical College of Hunan Normal University, Hunan Normal University, Changsha, 410008 China; 5 National Engineering and Research Center of Human Stem Cells, Changsha, 410008 China; 6 Key Laboratory of Stem Cells and Reproductive Engineering, Ministry of Health, Changsha, 410008 China

**Keywords:** CSE, Tissue engineering, Polymer materials, Three-dimensional structure, Stem cell, Regenerative medicine

## Abstract

Cell therapy is an accessible method for curing damaged organs or tissues. Yet, this approach is limited by the delivery efficiency of cell suspension injection. Over recent years, biological scaffolds have emerged as carriers of delivering therapeutic cells to the target sites. Although they can be regarded as revolutionary research output and promote the development of tissue engineering, the defect of biological scaffolds in repairing cell-dense tissues is apparent. Cell sheet engineering (CSE) is a novel technique that supports enzyme-free cell detachment in the shape of a sheet-like structure. Compared with the traditional method of enzymatic digestion, products harvested by this technique retain extracellular matrix (ECM) secreted by cells as well as cell-matrix and intercellular junctions established during in vitro culture. Herein, we discussed the current status and recent progress of CSE in basic research and clinical application by reviewing relevant articles that have been published, hoping to provide a reference for the development of CSE in the field of stem cells and regenerative medicine.

## Introduction

**Fig. 1:**
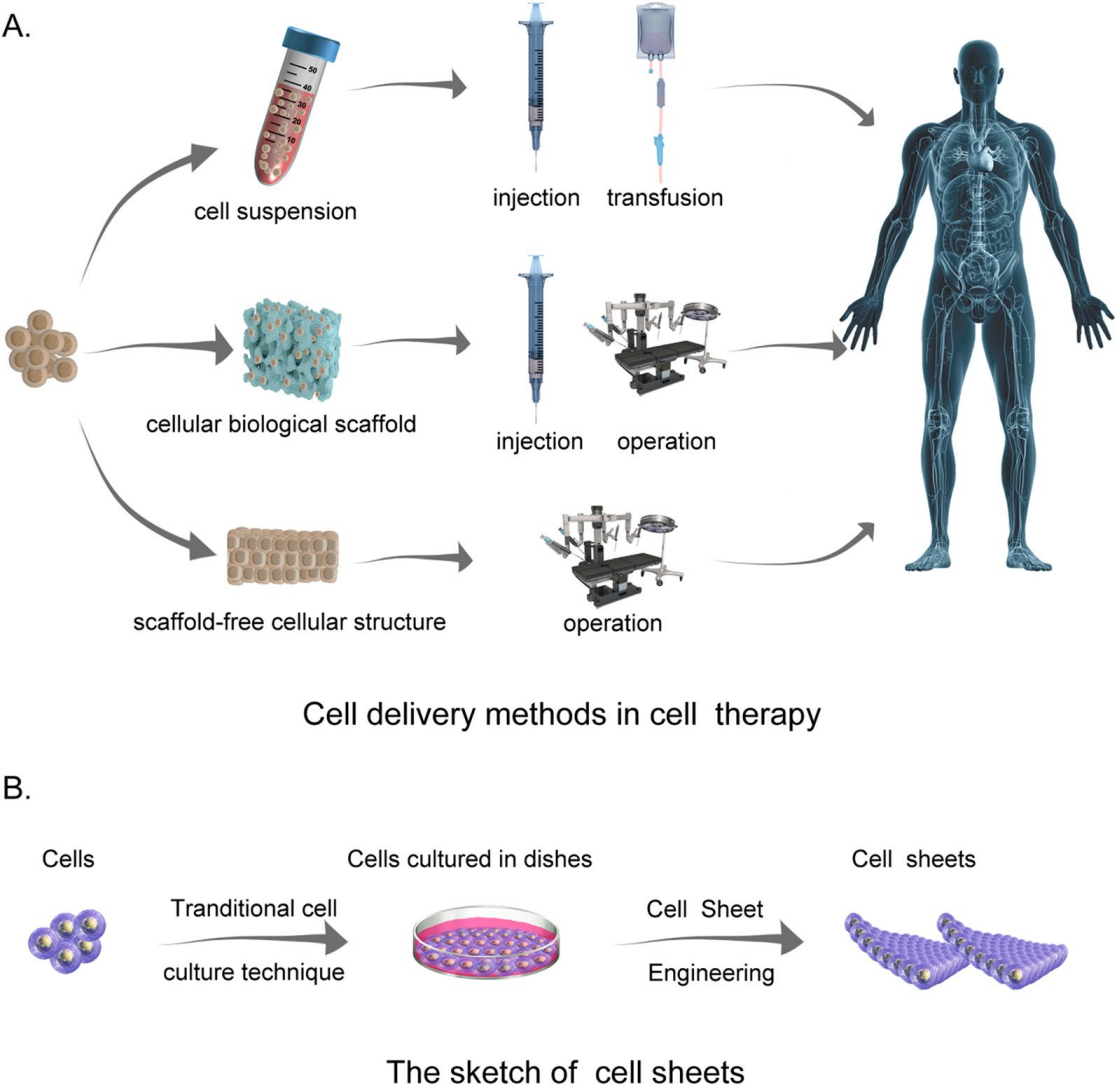
Schematic diagram of cell delivery methods in cell therapy (drawn by photoshop). **A** Three main cell delivery approaches are used in cell therapy. Some of the icons in this section are from AlexLMX/Getty Creative (https://www.vcg.com/creative/1199996796) and comotion_design|comotion_design/Getty Creative (https://www.vcg.com/creative/1002505480) and have already had copyright permission. **B** The sketch of cell sheets

Cell therapy has long been used in medicine [1]. In recent years, the rapid advancements in stem cell research and regenerative therapy have garnered much praise for paving the way to novel therapies [2], including anti-aging[3], cardiac repairs [4], the treatment of malignant tumors [5, 6], and end-stage liver disease [7]. Over recent years, this therapy has also been used to fight COVID-19 [8]. Unfortunately, although many trials have been performed, there is no consensus on the most optimal delivery strategy (some of the most popular methods are shown in Fig. 1A).

Cell suspension injection is a widely used cellular transplantation mode, mainly due to its convenience and ease of operation. This method has been applied to treat hypovolemic shock, hematological malignancies, and, more recently, COVID-19 [8–10]. However, it is unsuitable for cases requiring rebuilding a three-dimensional structure with a certain strength, such as osseous tissue repair [11].

Tissue engineering provides new solutions to tissue repair and regenerative medicine. This newly developed subject maintains, improves, and either restores tissue or entire organ’s structure and function by applying the principles of engineering science and life sciences to the development of biological substitutes. Two general protocols have been developed for biodegradable scaffold preparation: the first is a two-step process that contains the pre-production of a cell-free biodegradable scaffold and the seeding of functional cells[12]; the second is an only-one-step process that uses biomaterials to carry target cells and produces a cell-laden biodegradable scaffold [13]. In both protocols, the cells can grow and multiply with the degradation of scaffolds and finally, form a tissue-like structure that supports tissue replacement and repair.

However, intercellular interactions may be of great importance to the efficacy and mode of damage repair in some diseases. Once adherent cells are placed in suspension, their morphology, viability, and function may be affected. Therefore, the location and non-homogeneous distribution of the injected cells need to be controlled [14]. Moreover, using proteolytic enzymes such as trypsin for cell harvesting may degrade cell surface proteins simultaneously [15], which can affect the differentiation of injected cells and even result in the loss of their functions [16]. Conventional tissue engineering methods proved satisfactory in repairing tissues such as cartilage and bone, which consisted of relatively sparse cell structures and contained a large number of ECM. The structure formed during the reconstruction using biodegradable scaffolds may resemble its physiological structure [17]. Yet, biodegradable scaffolds cannot fully mimic cell density which matches the physiology requirements of tissues such as the heart or the liver (these organs consist of relatively dense parenchymal cells) [18]. More importantly, the non-specific inflammatory reactions of polymer materials after implantation are also noticed. Thus, it is significant and essential to explore new delivery devices and methods for cell therapies.

Scaffold-free tissue engineering is a rapidly developing technique in the field of tissue engineering. It allows the creation of cell-dense tissues that can be harvested as a sheet-like cell assembly without needing an enzyme (Fig. 1B) [19]. This technique pushes the limitations of traditional tissue engineering and allows the delivery of a higher number of cells to a target site [18]. Moreover, cell sheet transplantation avoids additional sutures because the preserved ECM and fibronectin can help the grafts adhere to the transplant site, thus mediating appropriate tissue regeneration and functional recovery [20, 21]. Herein, we discussed CSE's current status and recent progress in basic research and clinical application by reviewing relevant articles, hoping to provide a reference for the development of CSE in the field of stem cells and regenerative medicine.

## Cell sheet engineering—preclinical research

A number of preclinical studies have reported on emerging CSE technology for treating tissue or organ damage, like corneal injury, esophageal lesions, periodontosis, heart diseases, and liver diseases. This relatively new therapy has shown to be effective and generally safe in treating corresponding lesions in experimental animals, which provides favorable evidence for further clinical applications.

### Animal models

Animal models are a key factor in preclinical research. So far, CSE has been tested in mice, rats, dogs, rabbits, sheep, and swine [22–25]. Mice and rats are the most common model animals; they are easily accessible, have a high degree of homology to the human genome, have relatively short breeding cycles, have small cage requirements, and have low feeding costs [26]. Advantages of the rabbit and dog models compared to mouse and rat models include ease of surgery. On the other hand, pigs are an ideal animal model for human health and diseases because their anatomy and physiology are similar to humans [27]; yet, they are more difficult to maintain and have high feeding costs [27–29]. The efficiency and safety of cell sheets in cardiovascular diseases, esophageal diseases, and osteochondral defects have been proven in swine models [30–32]. Rabbit and dog models were also used in these diseases [33, 34], while rabbit models are more prominent in ophthalmic diseases such as limbal stem cell deficiency (LSCD) [35].

Cell sheets are transplanted into the body and need to remain in the host for a long time or become integrated with the host to exert therapeutic effects. Therefore, immunity and immunogenicity must be considered. Two major solutions have been proposed: one is to use cells with lower immunogenicity to fabricate cell sheets, like mesenchymal stem cells (MSCs), allogenic cells, or autologous cells [36–38]; the other is to use immunodeficiency animal models such as severe combined immunodeficiency (SCID) mice [39], Rag2-Il2rg double knockout mice [40], and nude rats [41]. As for big mammals, the immunodeficiency models are more difficult to establish and therefore, are unusual to be seen in reported research in the cell sheet field. Still, benefits from the development of transcription activator-like effector nuclease (TALENs) and CRISPR/Cas9 technology, there is some progress in immunodeficiency big mammals, which could be candidates in future preclinical studies [42], such as RAG 1- and 2-deficient rabbits [43], X-linked severe combined immunodeficiency (X-SCID) rabbits [44], RAG2 biallelic KO pig [45], and IL2Rγ KO pigs [46].

### CSE in the treatment of endocrine disease

Internal secretion is an essential function of keeping a healthy human organism, and the most common disorders in the endocrine department are diabetes and thyroid disease, both of which are targeted by cell sheet treatment.

#### Cell sheets in treating diabetes and its complications

Diabetes, a metabolic disease, is characterized by elevated levels of blood glucose caused by defective secretion of insulin and/or impaired biological action of insulin. Diabetes has been associated with certain cardiovascular events, diabetic kidney disease, diabetic retinopathy, and neuropathy [47]. Unfortunately, current conventional treatments for diabetes (especially insulin-deficient diabetes) remain insufficient in achieving long-term glycemic control.

Cell therapy for diabetes aims to restore endogenous insulin secretion, and CSE may have an advanced role in this therapy as a cell delivery method. In 2009, Shimizu et al*.* prepared rat islet cell sheets with temperature-responsive culture dishes and transplanted them into the subcutaneous space of recipient rats. Islet graft maintained the function of sensing and releasing insulin and reversed diabetes in streptozotocin-induced diabetic SCID mice [39]. Hirabaru et al*.* [48] seeded rat islets onto the converged MSCs, which were then transplanted into diabetes mellitus SCID mice. MSCs sheets showed a protective effect on maintaining islet function. Research in this field also indicated that MSCs could be replaced by other supporting cells like adipose tissue-derived stromal cells (ADSCs) and fibroblasts [49]. Supporting cell sheet in crafting engineered cell sheets composed of supporting cells and islets can facilitate islet viability and integration in the early stage of transplantation by secreting cytokines and providing abundant ECM, which means that they are better alternatives in islet transplantation.

Except for being transplanted into subcutaneous sites, alternative sites were exploited to break the limitation of implantation sites. The liver surface [50] and peritoneal wall [51] were also proven suitable for islet implantation. Meanwhile, islets generated from human chemically induced pluripotent stem cells (hCiPSC-islets), human MSCs, and human embryonic stem cells (hESCs) were also reported to be effective in restoring endogenous insulin secretion and improving glycemic control [52–54].

#### Cell sheets in treating thyroid dysfunction

The thyroid is an endocrine gland that has an important role in vital functions in maintaining normal reproduction, controlling fundamental physiological mechanisms, etc., by producing and secreting thyroid hormones [55]. Over the years, CSE has shown to be a valid alternative in the treatment of thyroid dysfunction. Arauchi et al*.* fabricated rat thyroid cell sheets for the first time and tested their efficiency in treating hypothyroidism by transplanting them into the subcutaneous site of rats that underwent total thyroidectomy. Results showed a typical thyroid follicle organization in the graft, and hypothyroidism caused by total thyroidectomy was recovered. Moreover, Huang et al*.* [56] obtained primary human thyroid cells from non-tumorous tissues of patients with thyroid cancer or Graves’ disease and successfully fabricated functional patient-specific non-tumorous thyroid cell sheets.

### CSE in the treatment of liver diseases

CSE has shown pre-clinical effectiveness and safety in treating liver diseases[57]. There have already been many reports on the fabrication of hepatic tissue sheets. Ohashi et al*.* [58] constructed vascularized liver tissues with various liver-specific functions by transplanting primary mice hepatocyte sheets into the vascularized subcutaneous cavity of wild-type FVB/N mice with the same background as the donor. Moreover, an engineered two-layer hepatocyte/fibroblast sheet (EHFSs) composed of a human primary hepatocyte sheet and a human diploid fibroblast TIG-118 cell sheet was reported in 2015 [59]. This one-step harvest EHFSs was transplanted into the abdominal subcutaneous site of immunodeficiency mice. The transplanted EHFSs established a vascular connection with the host and constructed subcutaneous organs with specific functions.

The liver tissue constructed by CSE has resemblant liver-specific functions as the primary liver, including protein secretion, the response to regenerative stimulation, and metabolic drug activity [60]. Besides, the vascularization and long-term viability of the engineered cell sheets in vivo have also been verified [61]. All these achievements have indicated the therapeutic potential of cell sheets in liver diseases and further facilitated their preclinical research in this field.

Baimakhanov et al*.* [62] transplanted multilayer rat Hep-fibroblast sheets into the subcutaneous site of allogenic liver failure rats who had received radiation irradiation and partial hepatectomy. The grafts vascularized and proliferated around them. Meanwhile, the survival rate and serum albumin concentration in the transplantation group were significantly higher than those in the control group, which suggested that the transplanted cell sheets provided support for liver metabolism and improved liver function. Furthermore, human induced pluripotent stem cells (hiPSCs) derived hepatocyte-like cell sheets were also proven to ameliorate liver function of carbon tetrachloride (CCl_4_) induced lethal liver failure mice by secreting hepatocyte growth factor [40]. In 2022, primary human hepatocytes (PHH) and hepatic stellate cells (HSC) were co-cultured in glucose and lipid-containing medium to form engineering cell sheets [63]. The hepatocytes in these cell sheets were similar to ballooned hepatocytes in human nonalcoholic steatohepatitis (NASH). Both of them showed ballooning, accumulation of fat droplets, abnormal cytokeratin arrangement, and the presence of Mallory–Denk bodies and abnormal organelles, indicating the co-cultured cell sheets of a new in vitro NASH model.

### CSE in the treatment of cardiovascular diseases

Cardiac tissue engineering is one of the most promising methods for treating severe heart failure [64]. Applying CSE technology to construct more complex and thicker vascularized soft tissues, curved surfaces, and hollow tissue structures is advantageous in curing cardiovascular diseases such as peripheral vascular disease and cardiomyopathies [65].

Monolayer human umbilical vein endothelial cells (HUVECs) sheets were reported to obtain great therapeutic efficiency in ischemic-reperfusion injury model mice [66]. In detail, the HUVECs sheets were rapidly prepared using multifunctional tetronic-tyramine hydrogels and then, transplanted to the injury site of the vessel ligation-induced ischemic hind limb mice. Compared with directly injected cell suspension, which contained a consistency of cells, the cell sheet was maintained longer at the target site and retarded tissue necrosis. Similar advantages of cell sheets were presented in dealing with ischemic cardiomyopathy model animals such as myocardial infarction rats [67, 68]. To establish a vascular connection with the host and obtain better therapeutic efficiency, endothelial lineage cells should be added to the engineered cell sheets. In addition, these endothelial networks in cell sheets were connected to the host blood vessel and perfused shortly after the transplantation of co-cultured cell sheets [69]. Except for endothelial lineage cells, stem cells such as MSCs and hiPSCs were reported to co-culture with cardiomyocytes (CMs). The proliferation of stem cells, expression of ETS1 (an endothelial differentiation marker), and Notch signaling activation were promoted via cell–cell interaction within cell sheets [70].

Kawamura et al*.* transplanted 7 iPSCs derived cardiomyocytes (hiPS-CMs) sheets in the epicardium of myocardial infarction mini-pigs. Their omentum was then placed over the grafts and fixed on the pericardium. As a result, the covered pedicled omentum flaps enhanced the viability of hiPS-CMs sheet grafts, and this unit prolonged the therapeutic effect of angiogenesis promotion, vascular maturation regulation, fibrosis reduction, and ventricular remodeling alleviation. Chang et al*.* [71] used human umbilical cord mesenchymal stem cells (hUC-MSCs) and thermally triggered cell-sheet fabrication techniques to construct cell sheets. And reccurenced the inspiring therapeutic effects in improving cardiac function of myocardial infarction(MI) porcine. Furthermore, Lu et al*.* [29] used cell-imprinting technology whose procedure was just like semiconservative replication to obtain a cell sheet. Cells in this kind of biomimetic cell sheet could be guided to realize the geometry tissue-imprinted biointerface, similar to the natural tissue morphology. The distribution and orientation of cells in this myocardium cell sheet were closer to the native pig heart tissue, with a stronger beating strength and longer lifetime. Also, this biomimetic cell sheet strategy provides us with new ideas for applications, such as in vitro construction of artificial organs.

### CSE in the treatment of ophthalmic diseases

With the continuous development of life science and biomaterials, CSE has made great progress in treating ophthalmic diseases. hiPSCs have been successfully differentiated into multiple ocular-like cell lineages and used to fabricate a variety of cell sheets. In addition, some pre-clinical efforts have been made to exploit the efficiency and safety of cell sheets such as corneal stem cell sheets [72], corneal epithelial cell sheets [73], oral mucosa cells (OMECs) sheets [74], hPSCs derived retinal pigment epithelium (RPE) sheets [75, 76], and MSCs sheets[77] in treating ophthalmic diseases.

Zhang et al*.* [27] used multilayered ESCs sheets to treat limbal stem cell deficiency (LSCD) model rabbits. The grafts on the corneal stroma experienced differentiation and facilitated the reconstruction of the corneal epithelial layer without conjunctival ingrowth or peripheral neovascularization. ADSCs-derived corneal epithelium [78] was used to form cell sheets with the help of N-isopropyl acrylamide-co-glycidyl methacrylate (NGMA)-coated substrates. These cell sheets were then spread over the corneal surface that was hurt by n-heptanol. Also, this was indicative of injury concrescences as well as reconstruction effects on the ocular surface.

The RPE-related lesion is another important component of ophthalmic diseases. To solve a series of RPE deficiencies like retinal degeneration, hPSCs such as hiPSCs [76] and hESCs [75] have been induced into RPE and fabricated hPSCs-RPE sheets which have long-term integration capacity in vivo and feasibility of surgical transplantation in clinical application [79]. These engineered cell sheets expressed typical RPE markers, patterns of gene expression, polarized secretion of growth factors, and similar phagocytotic capacity to native RPE [76].

### CSE in the treatment of oral and periodontal diseases

The regeneration of oral and periodontal tissues requires a highly coordinated spatiotemporal healing response; thus, its treatment is extremely challenging. However, CSE is conducive to regenerating and repairing oral and periodontal tissues [80], which promises a new method for treating oral and periodontal diseases.

Hu et al*.* [81] formed multilayer human dental pulp stem cells (hDPSCs) sheets with the supply of Vc, which were then transplanted to the bone defect location of the periodontitis model swine [82], showing stronger bone regeneration capacity compared to cell suspension injection. Meng et al*.* [83] created hTDM/hDPSCs complex by seeding hDPSCs onto human dentin matrix (hTDM), which can significantly up-regulate the mRNA expressions of dentin sialophosphoprotein (DSPP), osteocalcin (OCN), and vascular endothelial growth factor receptor 1 (VEGFR1) in hDPSCs [84]. Then, a mixture of matrigel and hDPSCs was injected into the complex cavity. Finally, this prepared construction was wrapped up using a Vc-induced hDPSCs sheet. The ability of this sandwich structure to regenerate tooth roots was demonstrated by implanting it into the dorsum of mice. Another study used temperature-responsive culture plates to construct complex cell sheets containing approximately 10 layers of rat periodontal ligament cells (PDLCs) and osteoblasts (MC3T3-E1), testifying to the regenerative ability via heterotopic transplantation [85].

CSE also has great potential in treating cleft palate, canker sores, and xerostomia. Nam et al*.* [86] performed in vitro and in vivo studies of submandibular gland (SMG) cell sheets. In vitro study results showed that monolayer SGM sheets could maintain secretory granules and cell–cell junctions, while double-layer cell sheets had a glandular-like pattern. Furthermore, double-layer SMG sheets had a stronger agonist-mediated response and a promotion of salivary gland repair. Furthermore, Lee et al*.* [87] fabricated MSCs sheets and observed a good curative effect in a rabbit model of chemically induced canker sores. The implanted cell sheet facilitated the complete coverage of basal cells and full-thickness mucosa healing in the ulcer region. They also assessed the effect of this cell model on repairing the cleft palate and attempted to search for the possibility of regenerating palatal bone with engineered cell sheets containing hMSCs and stem cells from human exfoliated deciduous teeth (SHEDs)[88].

### CSE in skin wound healing and tissue defect repair

At present, cell sheets prepared from ADSCs [89], human keratinocyte and fibroblast cells [90], and OMECs [91, 92] have been widely used in the study of skin wound healing and tissue defect repair, providing a feasible novelty method for the treatment of these diseases [93]. For example, autologous adipose-derived stromal cells (ASCs) sheets were implanted into the skin defects area in rats or mice [94, 95] to exploit the benefit of CSE in wound healing. The grafts could significantly enhance cell proliferation and angiogenesis, reduce the inflammatory response, and accelerate full-thickness wound healing via collagen accumulation [96]. Other studies compared cell sheet transplantation with traditional split-thickness skin grafts (STSGs) transplantation. Briefly, HUVECs and hMSCs were co-cultured to prepare pre-vascularized hMSC cell sheets (PHCS)[97], which were then plated onto the full-thickness excisional wound site [98]. Evaluations of wound healing effects of these grafts showed that PHCSs transplantation was superior in reducing skin scar contracture and improving cosmetic appearance [99].

The therapeutic efficiency of cell sheets in deep soft tissue defects was also proven in a splinted defect rat model [100]. To investigate the therapeutic efficiency of MSCs sheets and MSCs suspension, they were separately delivered to the injury site of splinted defect rats whose trapezius muscle defect was induced by excising skin-fascial flap on the back. The wound healing rate was tested, MSCs sheets-grafting has shown earlier granulation tissue formation and fibrosis occur, indicating that they had a higher healing efficiency in promoting defect contraction and scar formation.

Artificial ulcers, bleeding, and ulcerative esophageal contracture are common adverse events of endoscopic submucosal dissection (ESD)[101]. Over the years, several therapeutic methods have been proposed to prevent these events, including CSE. Ohki et al*.* developed a novel therapy combining ESD with OMSCs sheet transplantation [28]. This method was tested in male beagle dogs after receiving ESD. Briefly, accelerated ulceration healing and reduced scar formation were observed after transplantation. Furthermore, the mature epithelium of the grafts was intact, stratified, and resembled the native esophagus surface, indicating that cell sheets grafts can reduce inflammation and prevent complications after ESD [28]. Besides, the effect of allogeneic transplantation of ADSCs sheets [102] and epidermal cell sheets [32] in preventing ulcerative esophageal contracture using ESD-induced artificial esophageal ulceration model pigs has also been verified.

Pressure ulcers are usually caused when an area of skin is placed under pressure. People with impaired mobility or sensation, especially elderly patients undergoing natural skin changes with aging, are at the highest risk of pressure ulcers [103]. Moreover, patients with neurologic impairment (for instance, spinal cord injury), sedation, and peri-or postoperative immobilization are more likely to develop this kind of ulcer [104, 105].

Cell therapy has already shown improved rates of wound repair in pressure ulcers [106, 107]. Yet, delivery methods of therapeutic cells and their rapid loss in the wound bed limit their clinical application [108]. Therefore, CSE has become a selection of advantages. Alexandrushkina et al*.* [109] compared the efficiency of inducing healing in cutaneous pressure ulcers between adipose-derived mesenchymal stromal cell injection and adipose-derived mesenchymal stromal cell sheets transplantation. The results showed that adipose-derived mesenchymal stromal cell sheets could induce the recovery of dermal structure and its appendages, leading to obvious hair growth in pressure sores and their histological sites. On the other side, the retention of cell sheets was only 3–7 days. Before the rejection of CS, they fell off along with scab by natural detachment or during removal of wound dressing; however, this did not affect the treatment efficiency of CS for PU [110, 111]. The rapid elimination of cell sheets means lower risk tumorigenesis is conducive to clinical safety.

In addition, the applications of cell sheet transplantation in strengthening the anastomotic strength of intestinal anastomosis [112], enhancing healing of biliary anastomosis[113], and preventing pancreatic fistula in pancreatic surgery[114] have also been reported. Some researchers found that applying ADSCs sheets [113] and myoblast sheets [114] into the anastomosis area or pancreas section may promote pancreatic fistula and anastomosis healing.

### CSE in the treatment of some other diseases

In addition to the extensive research on cardiovascular, liver, skin, and mucosa, the potential of cell sheets for the urinary system, motor system, and reproductive system has also been explored. In the motor system, repair and functional recovery of the rotator cuff in rotator cuff tears [115], as well as regeneration and repair of tendon and/or ligament in tendon injuries [116], remain challenging. CSE has been recommended as an effective alternative approach for preventing fibrovascular scar formation and improving specific functions during healing. Liu et al*.* [117] introduced a novel biomaterial constructing engineered tendon-fibrocartilage-bone composite (TFBC) and BMSCs sheets, which positively affects rotator cuff repairs of acute and full-thickness tendon injury in a canine non-weight-bearing model. Besides, there have been many attempts to apply CSE in cartilage repair and bone regeneration. Osteochondral defects repair of various cell sheets such as chondrocyte sheets [30], human amniotic mesenchymal stem cells (*h*AMSCs) sheets [118], ASCs-HUVECs-ASCs triple-layer sheets [119], frozen/fresh osteoblasts induced by gelatin membrane (FT/F—GCS) [120] have already been reported, thus having a promising clinical application potential in motor system injuries.

End-stage kidney disease, including diabetic nephropathy, is a serious life-threatening condition difficult to treat with traditional therapies [121]. Several studies have suggested using cell therapy to treat these diseases [122]. Takemura et al*.* [123]found that transplantation of ASCs sheets may improve engraftment efficiency and retard the progress of renal injury in diabetic nephropathy model rats. Moreover, powerful therapeutic effects of hepatocyte growth factor (HGF)—secreting mesothelial cell sheets [124] and BMSCs sheets [125] were reported in a rat model of chronic kidney disease(CKD). The grafts with a higher survival rate reduced the degree of renal fibrosis and renal microvascular injury, thus providing a great alternative for treating renal failure and its complications [126].

As an attractive resource of cell therapy, engineered cell sheets are also showing promising effects in liver-related genetically coded diseases like hemophilia caused by a deficiency of coagulation factors. Hemophiliacs are generally treated with an expensive protein replacement treatment relying on a periodical supplement of coagulation factors [127]. The fact that only a tiny amount of coagulation factors can maintain normal function makes it suitable for cell therapy with CSE to fit the treatment of hemophilia as it helps to resolve the difficulty of cells’ long-term maintenance and vascularized integration. Watanabe et al*.* [128] performed lentiviral vector transduction on murine ADSCs to endow them with the ability of human coagulation factor IX (hFIX) production. The fabricated genetically modified ADSCs sheets, which could secret hFIX showed a novel choice in treating Hemophilia B. A similar operation was performed in blood outgrowth endothelial cells (BOECs) isolated from mice with hemophilia A. The results of in vitro/in vivo analysis indicated that cell sheet implantation acquired a much better integration capacity than BOECs- Matrigel complexus and had a Long-term effect in treatment [129].

Endometrial cell sheets may also express specific markers, such as female-specific hormone receptors. These cells can be fabricated to reconstruct endometrium-like tissue in nude rats [130]. Furthermore, further studies of cell sheets in preventing intrauterine adhesions after endometrial injury [131], as well as improving uterine incision repair [132] and facilitating fertilization and pregnancy in endometrial defect model rats [133], were reported. Altogether, the CSE technique has great advantages in regenerating endometria and might be a novel method for endometrial disorders treatment with huge potential.

### Brief summary

In recent years, scaffold-free CSE has shown promising potential in tissue regeneration. This technology has been applied to several cell types, including BMSCs, ADSCs, thyroid cells, hepatocytes, fibroblast, PHHs, and HSCs. More recently, HUVECs, CMs, corneal epithelial cells, OMECs, and hDPSCs, together with cell sheet engineering, have already been reported to have an important part in tissue reconstruction and repair. Directed induction of functional cells from stem cells (either hESCs or hiPSCs) has shown to be a promising alternative for the reconstruction of dysfunction. stem-cell-derived CMs, stem-cell-derived hepatocyte-like cells, stem-cell-derived RPE and others are all virtually endless sources of cell sheets engineering. Preclinical studies have shown that cell sheets engineering plus cell therapy could be a promising, safe, and effective method for treating most types of body conditions.

## Cell sheet engineering-clinical trials

ECM layer, a kind of glue preserved in CSE, ensures cell sheets tightly adhere to the target of the host without using artificial scaffolds or conducting additional operations such as suturing [134]. At present, the application research of CSE in the medical field has shifted from basic, preclinical research to clinical trials. In the next paragraph, we will provide recent updates in the field.

### Registered clinical trials of CSE

**Fig. 2:**
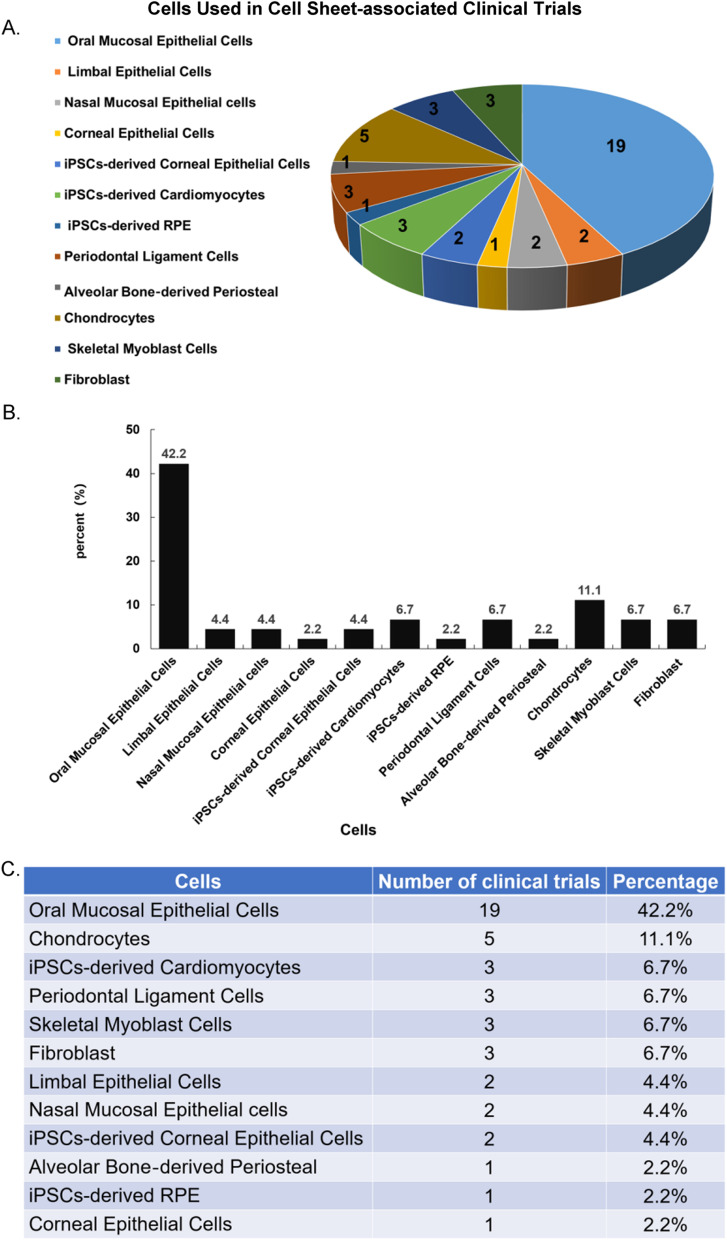
Cell resources used in 45 clinical trials assessing cell sheets-associated clinical trials. **A** The chart shows a pie diagram of the 12 kinds of cells used in cell sheet-associated clinical trials; the number of clinical trials for each cell type. **B** Bar graph indicating the percentage of each cell type used in cell sheet-associated clinical trials. **C** Table with 12 cell types and their corresponding number of clinical trials and percentage among the 45 clinical trials

We retrieved the clinical trials registry database of the International Clinical Trials Registry Platform (ICTRP) and summarized the clinical trials related to cell sheet therapy (excluding termination status) published until September 2022 (Table 1). A total of 45 clinical trials have been found, most of which focused on the esophageal mucosa and ocular surface lesions, which lack effective treatment methods. Also, the preferred cell resources are oral mucosa cells (Fig. 2). Because oral mucosa cells are non-keratinization, hair-free, anti-infection, and promoting wound regeneration [135, 136]. Moreover, compared with other types of cells, oral mucosal cells are easier to obtain [137].

**Table 1:** Clinical trials that are relevant with cell sheets

Title	Identifier	Recruitment status	Condition or disease	Materials	Phase/basic objective	Participants
Phase I study to evaluate safety of LSCD101(cultured autologous limbal epithelial cell sheet) transplantation for limbal stem cell deficiency	NCT04773431	Completed	Limbus corneae/limbus corneae insufficiency syndrome	LSCD101 (cultured autologous limbal epithelial cell sheet)	Safety	6
Clinical trial of human (allogeneic) iPS cell-derived cardiomyocytes sheet for ischemic cardiomyopathy	NCT04696328	Recruiting	Myocardial ischemia	Human (allogeneic) iPSCs derived-cardiomyocyte sheet	Safety efficacy	10
Safety and efficacy study of cells sheet-autologous chondrocyte implantation to treat articular cartilage defects (CS-ACI)	NCT01694823	Recruiting	Osteochondritis/osteochondritis dissecans/joint diseases	Cells sheet-autologous chondrocyte (CS-AC)	Safety efficacy	10
autologous cell sheets transplantation after ESD in the esophagus	NCT02455648	Completed	Barrett esophagus	Autologous oral mucosal epithelial cell sheet	Efficacy	10
Transplantation of autologous oral mucosal epithelial stem cell sheet for treating limbal stem cell deficiency disease	NCT03015779	Enrolling by invitation	Limbal stem cell deficiency	Cultured autologous oral mucosal epithelial cell sheet	Efficacy	7
Human (autologous) oral mucosal cell sheet transplantation after ESD in patients with superficial esophageal cancer	NCT02866019	Completed	Superficial esophageal cancer	Human (autologous) oral mucosal cell sheets (CLS2702C/CLS2702D)	Safety efficacy	10
Cultured autologous oral mucosa epithelial sheet for the treatment of bilateral limbal stem cell deficiency	NCT03949881	Recruiting	Total bilateral limbal cell deficiency	Autologous oral mucosa epithelial sheet (autologous jugal mucosa cell sheet, FEMJA)	Tolerance efficacy	40
Clinical trial on the effect of autologous oral mucosal epithelial sheet transplantation	NCT02149732	Available	Limbal stem cell deficiency/Stevens–Johnson syndrome/ocular cicatricial pemphigoid/chemical burn	Cultured autologous oral mucosal epithelial sheet	Efficacy	?
Transplantation of autologous oral mucosal epithelial sheets for limbal stem-cell deficiency	NCT02415218	Completed	Limbal stem-cell deficiency	Autologous oral mucosal epithelial sheets	Safety efficacy	6
Periodontal ligament stem cell implantation in the treatment of periodontitis	NCT01082822	Active, not recruiting	Chronic periodontitis	Periodontal ligament stem cell (PDLSC) sheet	Safety efficacy	80
Implement of autologous skeletal myoblast cell sheet transplantation for dilated cardio myopathy aiming precision medicine (implement of autologous skeletal myoblast cell sheet transplantation for dilated cardiomyopathy aiming precision medicine)	JPRN-jRCTb050200131	Recruiting	Dilated cardiomyopathy	Autologous skeletal myoblast cell sheet	Safety efficacy	4
Multicenter trial of autologous nasal mucosal epithelial cell sheets for chronic middle ear inflammatory disease (middle ear cholesteatoma)	JPRN-jRCT2033210438	Recruiting	Chronic middle ear inflammatory disease (middle ear cholesteatoma)	Autologous nasal mucosal epithelial sheets	Safety efficacy	12
Clinical trial of human (allogeneic) iPS cell-derived cardiomyocytes sheet for ischemic cardiomyopathy (Follow-up trial)	JPRN-jRCT2053220055	Recruiting	D017202 Ischemic cardiomyopathy	iPSCs-derived cardiomyocytes sheet	Safety efficacy	10
First-in-human clinical research of iPS derived corneal epithelial cell sheet transplantation for patients with limbal stem-cell deficiency	UMIN000036539	Complete: follow-up continuing	Limbal stem-cell deficiency	iPSCs-derived corneal epithelial cell sheet (iCEPS)	Safety efficacy	4
Long-term follow-up of the multicenter investigator-initiated clinical trial using cultivated autologous oral mucosal epithelial cell sheet (COMET01) transplantation for patients with limbal stem-cell deficiency	UMIN000039992	Complete: follow-up complete	Limbal stem-cell deficiency (LSCD)	Autologous oral mucosal epithelial cell sheet (COMET01)	Safety efficacy	6
Clinical trial of human (allogeneic) induced pluripotent stem cell-derived cardiomyocyte sheet for severe cardiomyopathy	UMIN000032989	Complete: follow-up complete	Ischemic cardiomyopathy	Human (allogeneic) iPSCs-derived cardiomyocyte sheet	Safety efficacy	3
Clinical trial of autologous oral mucosal epidermal cell sheet transplantation in patients with anastomotic re-stricture after repair of congenital esophageal atresia	UMIN000034566	Recruiting	Congenital esophageal atresia	Autologous oral mucosal epidermal cell sheet	Safety efficacy	5
A phase I/IIa safety and efficacy study of allogeneic periodontal ligament-derived mesenchymal stromal cell sheet product (TWP-0001) in patients with periodontitis, consisting of 1 wall intrabony defect, Class III furcation defect, or horizontal defect, with probing depths of 4–9 mm after the initial therapy	UMIN000034310/ JMA-IIA00391	Complete: follow-up continuing	Periodontitis	Allogeneic periodontal ligament-derived mesenchymal stromal cell sheet (TWP-0001, periodontal ligament stem cell sheet)	Safety efficacy	6
Clinical trial related with transplantation therapy with human autologous cell mixed sheets for refractory cutaneous ulcer	UMIN000031645	Recruiting	Refractory cutaneous ulcer	human autologous cell mixed cell sheet (fibroblasts and peripheral blood mononuclear cells)	Safety	6
Sealing of lung air leaks by fibroblast sheets	UMIN000022554	Complete: follow-up complete	Pneumothorax, bullae, benign lung tumors	Autologous fibroblast sheets	Safety	5
Tissue-engineered cultured periosteum used with platelet-rich plasma and hydroxyapatite in treating human osseous defects: second phase of clinical trial	UMIN000018788	Complete: follow-up complete	Chronic periodontitis	Cultured alveolar bone‐derived periosteal sheet	Safety efficacy	30
Multicenter Investigator-initiated clinical trial using cultivated autologous oral mucosal epithelial cell sheet (COMET01) transplantation for patients with limbal stem-cell deficiency	UMIN000018662	Complete: follow-up complete	Limbal stem-cell deficiency	Autologous oral mucosal epithelial cell sheet (COMET01)	Efficacy	6
Autologous myoblast sheets transplantation therapy for cardiomyopathy	UMIN000012906	Complete: follow-up continuing	Dilated cardiomyopathy, Ischemic cardiomyopathy	Autologous skeletal myoblast sheet	Safety efficacy	40
Clinical trial of cultivated autologous oral mucosal epithelial cell sheet transplantation for corneal epithelial stem cell deficiency	UMIN000012264	Complete: follow-up continuing	Corneal epithelial stem cell deficiency	Autologous oral mucosal epithelial cell sheet	Safety efficacy	10
Clinical trial of cultivated autologous oral mucosal epithelial cell sheet transplantation for corneal epithelial stem cell deficiency	UMIN000012260	Complete: follow-up complete	Corneal epithelial stem cell deficiency	Autologous oral mucosal epithelial cell sheet	Safety efficacy	10
Safety and efficacy of transplantation of oral mucosal epithelial cell sheets in preventing formation of strictures after ESD	UMIN000010251	Complete: follow-up complete	Superficial esophageal cancer	Autologous oral mucosal epithelial cell sheet	Safety efficacy	10
A study of transplantation of autologous induced pluripotent stem cell (iPSC) derived retinal pigment epithelium (RPE) cell sheet in subjects with exudative age-related macular degeneration	UMIN000011929	Complete: follow-up complete	Exudative age-related macular degeneration (AMD)	Autologous iPSCs-derived RPE sheets	Safety	6
Safety and Efficacy of Autologous Myoblast Sheets Treatment for Patients with Severe Heart Failure due to Chronic ischemic Heart Disease	UMIN000008013	Complete: follow-up complete	Severe heart failure due to chronic ischemic heart disease	Autologous skeletal myoblast sheet	Safety efficacy	6
Clinical study of the autologous oral mucosal epithelial cell sheets in preventing formation of strictures after endoscopic submucosal dissection for esophageal cancer	UMIN000026506	Recruiting	Esophageal stricture after ESD	Autologous oral mucosal epithelial cell sheet	Safety	6
The clinical study for the joint treatment by allogeneic cell sheet	UMIN000015205	Complete: follow-up continuing	Cartilage defects associated with osteoarthritis of the knee	Chondrocyte sheet	Safety efficacy	10
Clinical trial of cultivated autologous oral mucosal epithelial cell sheet transplantation for corneal epithelial stem cell deficiency	UMIN000006745	Complete: follow-up complete	Corneal epithelial stem cell deficiency	Autologous oral mucosal epithelial cell sheet	Safety efficacy	10
The clinical study for the joint treatment by cell sheet	UMIN000006650	Complete: follow-up complete	Traumatic and/or degenerative cartilage defect of the knee joint	Autologous chondrocyte sheets	Efficacy	10
Development of new strategy for severe heart failure using autologous myoblast sheets	UMIN000003273	Complete: follow-up complete	Ischemic cardiomyopathy, dilated cardiomyopathy	Autologous skeletal myoblast sheet	Safety efficacy	16
Periodontal regeneration with autologous periodontal ligament cell sheets	UMIN000005027	complete	Periodontitis	Autologous periodontal ligament cell sheets	Safety efficacy	10
Cultivated epithelial sheet transplantation for corneal epithelial stem cell deficiency	UMIN000002948	Complete: follow-up complete	Corneal epithelial stem cell deficiency	Corneal epithelial sheet	Safety efficacy	5
Clinical trial of cultivated autologous oral mucosal epithelial cell sheet transplantation for corneal epithelial stem cell deficiency	UMIN000005400	Complete: follow-up complete	Bilateral corneal epithelial stem cell deficiency	Autologous oral mucosal epithelial cell sheet	Safety efficacy	10
Treatment of artificial esophageal ulcerations after EMR by endoscopic transplantation of autologous oral mucosal epithelial cell sheets	UMIN000000473	Complete: follow-up complete	Artificial esophageal ulcerations after EMR	Autologous oral mucosal epithelial cell sheet	Safety efficacy	10
Clinical study of the autologous oral mucosal epithelial cell sheets in preventing formation of strictures after endoscopic submucosal dissection for esophageal cancer	UMIN000038051	Complete: follow-up complete	Esophageal narrowing after ESD	Autologous oral mucosal epithelial cell sheet	Safety	6
Phase I Study to Evaluate Safety of LSCD101(cultured autologous limbal epithelial cell sheet) transplantation for limbal stem cell deficiency	KCT0004741	Recruiting	Diseases of the eye and adnexa	LSCD101 (cultured autologous limbal epithelial cell sheet)	Safety	6
Clinical study of the autologous oral mucosal epithelial cell sheets in preventing formation of strictures after endoscopic submucosal dissection for esophageal cancer	jRCTb070190053	Recruiting	Esophageal stricture after ESD	Autologous oral mucosal epithelial cell sheet	Safety	6
Clinical study on the regenerative therapy for articular cartilage using autologous cell sheets	jRCTb030190166	Recruiting	Cartilage defects associated with osteoarthritis of the knee	Autologous chondrocyte sheet	Safety efficacy	20
A Study to Evaluate the Esophageal Stenosis Inhibition Effects of CLS2702C/CLS2702D After Endoscopic Submucosal Dissection (ESD) in Patients With Superficial Esophageal Cancer in the steroid administration risk group	jRCT2063200048	Recruiting	Superficial esophageal cancer after ESD	Human (autologous) oral mucosal cell sheets (CLS2702C/CLS2702D)	Efficacy	17
Realization for regenerative therapy of middle ear mucosa by using autologous nasal mucosal epithelial cell sheets	JMA-IIA00316	Completed	Middle ear cholesteatoma, adhesive otitis media	Autologous nasal mucosal epithelial sheets	Safety efficacy	10
First-in-human clinical research of iPS derived corneal epithelial cell sheet transplantation for patients with limbal stem-cell deficiency	jRCTa050190084	Not Recruiting	Limbal stem-cell deficiency	iPSCs-derived corneal epithelial cell sheet	Safety efficacy	4
Autologous cell mixed sheets transplantation for refractory cutaneous ulcer	jRCTb060190034	Recruiting	Venous skin ulcer	Human autologous cell mixed cell sheet (fibroblasts and peripheral blood mononuclear cells)	Safety efficacy	6

**Fig. 3:**
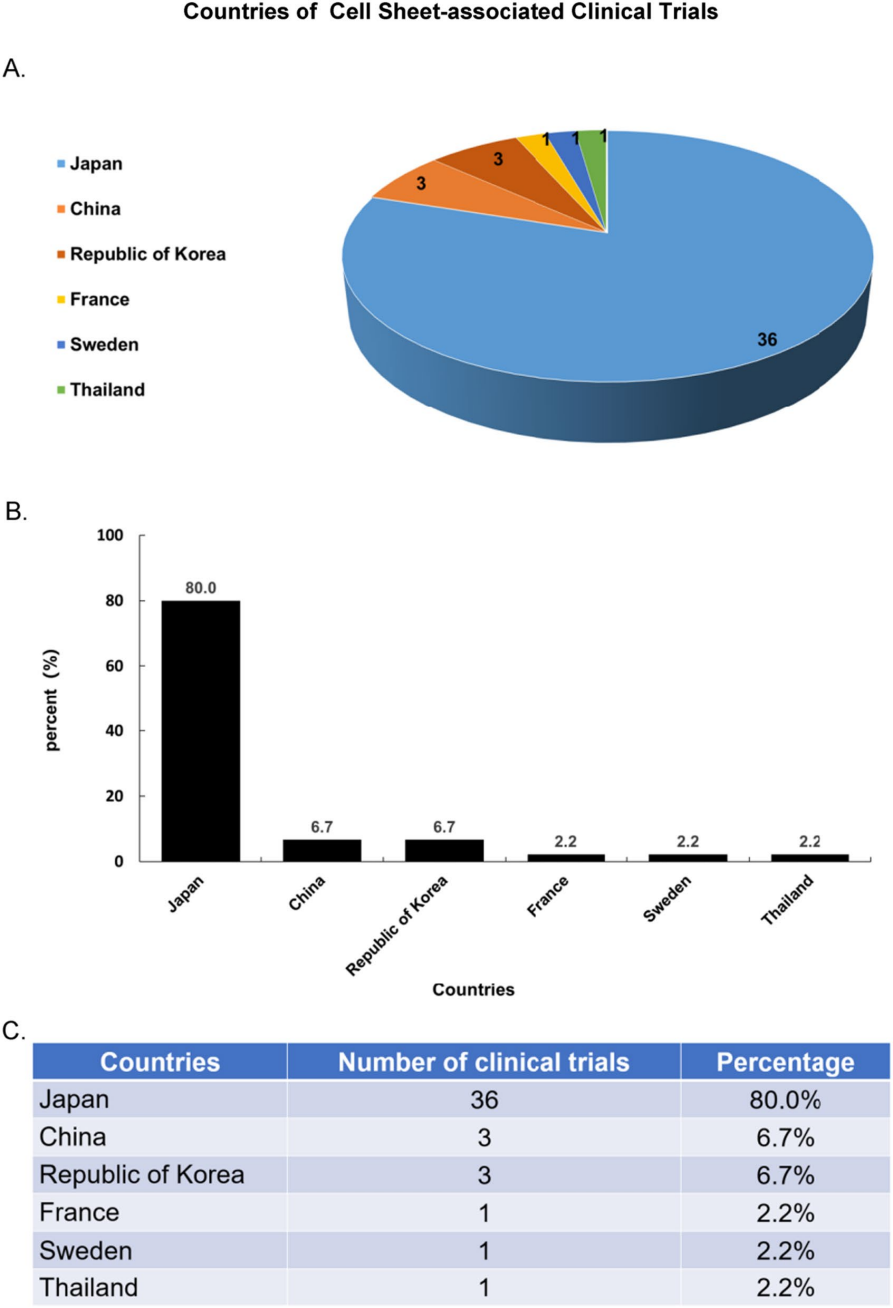
Major contributors in the field. **A** Japan has made the greatest progress in the field of CSE and iPSCs research, followed by China, the Republic of Korea, France, Sweden, and Thailand. Also, the number of registered clinical trials in each country was noted on the chart. **B**, **C** The bar graph indicates that 80% of trials have been registered in Japan. China and the Republic of Korea ranked second

**Fig. 4:**
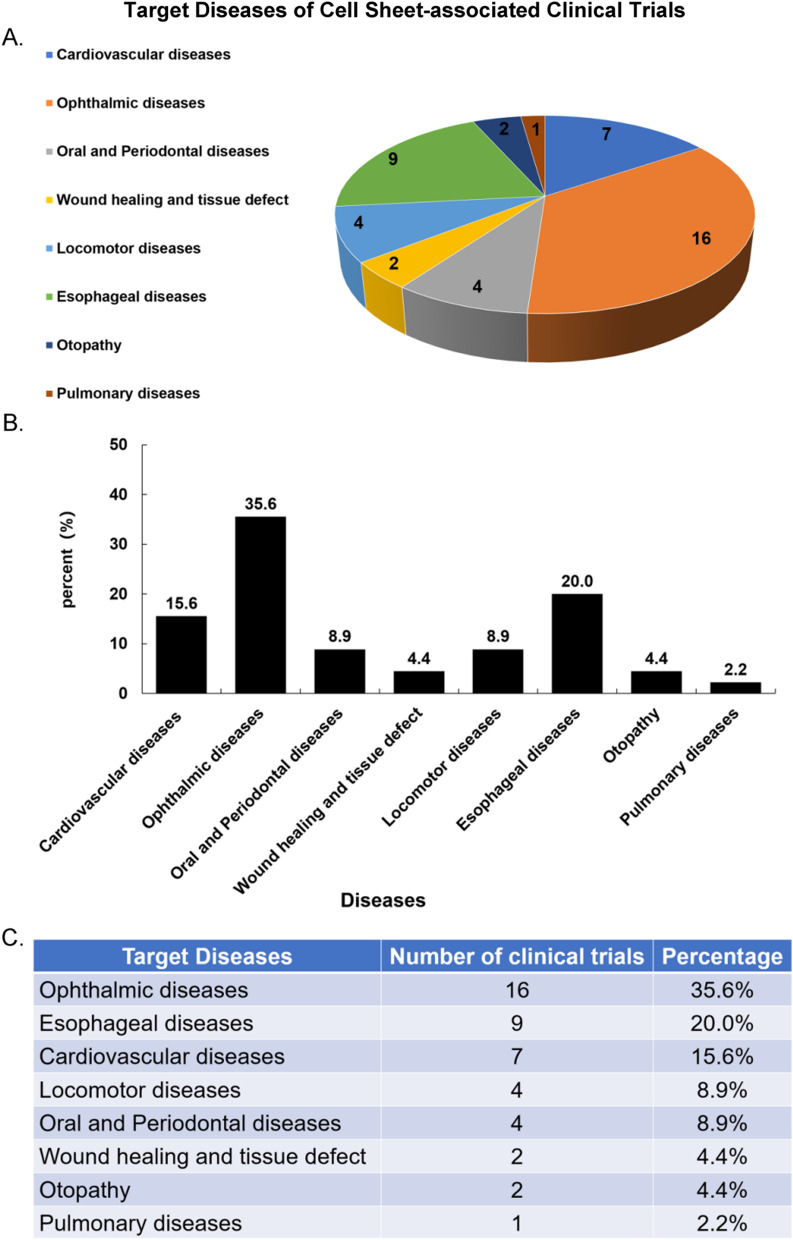
Aimed condition or diseases of the 45 registered cell sheets-associated clinical trials. **A** The aimed condition or diseases of these clinical trials were ophthalmic diseases, esophageal diseases, cardiovascular diseases, locomotor diseases, oral and periodontal diseases, and so on. **B**, **C** The proportion of each target disease in registered clinical trial

Japan has made the greatest progress in CSE and iPSCs research. More than 80% of the clinical trials related to cell sheets have been registered by Japanese scientists (Fig. 3). CSE has been mostly used to treat ophthalmic diseases, including Limbus Corneae Insufficiency Syndrome, Limbal Stem Cell Deficiency, age-related macular degeneration (AMD) et al. [138, 139] (16 of 45) of registered clinical trials. Other studies included esophageal diseases and cardiovascular diseases (Fig. 4).

### Reported clinical application of CSE

Some researchers have explored clinical applications of CSE and reported the results of these clinical trials.

#### Clinical trials of cell sheets in esophageal diseases

Ohki et al*.* [140] prepared cultured oral mucosa cells (COMECs) sheets from 9 patients who suffered from superficial esophageal neoplasms and then, transplanted these autologous epithelial cell sheets onto the ulcer surface through endoscopes when esophageal lesions were removed. In the follow-up and assessment, they reported the integration of the grafts and their preventive effects on scar formation and esophageal stenosis. Besides, patient-specific cell sheets were safe when transported by air [141]. These results indicate that cell sheet grafts promote the reepithelization of esophageal safely and effectively after ESD, improving the living quality of these patients [142]. Thus, CSE should be regarded as a safe and promising regenerative medicine technology.

#### Clinical trials of cell sheets in ocular surface diseases and retinal diseases

Nishida et al*.* reported the transplantation of autologous COMECs sheets in 4 patients with bilateral total corneal stem-cell deficiencies, confirming that the transplantation of cell sheets without suturing can be used to reconstruct the corneal surface and restore the vision of patients with bilateral severe ocular surface diseases. Since then, researchers [139, 143] have attempted to treat patients with limbal epithelial stem cell deficiency (NCT02149732) using autologous oral mucosa epithelial cell sheets. They evaluated the safety and efficacy of cell sheet transplantation according to adverse events and composite criteria, including the unepithelialized cornea proportion, the epithelialized cornea proportion, improved visual acuity, corneal conjunctival epithelium, number of vascular pedicles, and vascular activity, etc. The results showed that COMECs sheet transplantation is an effective and safe method for ocular surface reconstruction in patients with ocular surface diseases. A study (NCT02415218) published by Mahidol University published in 2022 reported transplanting autologous cultivated oral mucosal cell sheets onto the cornea of these patients’ affected eyes. The results indicated that cell sheets transplantation could improve visual acuities and decrease vascularization and epithelial defect.

In addition to treating ocular surface diseases, CSE also provides a breakthrough for retinal regeneration. Takahashi's research team has successfully prepared a single layer of RPE (hiPSC-RPE) sheet that can meet the clinical needs [76] and evaluate the effect of the autologous hiPSC-RPE sheet on age-related macular degeneration (AMD) in clinical trials [144] (UMIN000011929). They found that the grafts had satisfied integrity after transplantation, and the enlargement of pigmented colonies was similar to that in a study (NCT01345006) on the treatment of AMD with hESCs-RPE suspension injection reported in 2015 [145], which suggested that some photoreceptor cells have been regenerated and recovered.

#### Clinical trials of cell sheets in cardiac diseases

In 2012, Sawa et al*.* [146] first reported a significant improvement in clinical condition in a 56-year-old male patient with dilated cardiomyopathy (DCM) after receiving autologous myoblast cell sheet transplantation. This patient discontinued a left ventricular assist system (LVAS) after transplantation and did not develop life-threatening arrhythmia. Thereafter, the same team [147] evaluated the safety and efficacy of autologous bone myoblast sheets (TCD-51073) in the treatment of heart failure caused by severe ischemic heart disease through clinical trials in 7 patients with severe ischemic heart disease and chronic heart failure (Registration Number: UMIN000008013). With the changes in New York Heart Association (NYHA) class, specific activity scale (SAS), and echocardiographic parameters such as left ventricular ejection fraction (LVEF), it was believed that TCD-51073 could maintain and improve cardiac function, demonstrating the feasibility and safety of TCD-51073 transplantation in patients with severe ischemic heart disease and chronic heart failure.

Another study (Registration Number: UMIN000003273) isolated autologous skeletal muscle cells from 15 patients with ischemic cardiomyopathy and 12 patients with dilated cardiomyopathy to prepare cell sheets [148], confirming the safety and feasibility of cell sheets in the treatment of severe congestive heart failure. However, not until recently has the incidence of left ventricular (LV) recovery and the long-term outcomes following cell sheet transplantation been clarified (Registration Number: UMIN000012906) [149], and this latest report declared that 70% of ischemic cardiomyopathy patients benefit long-term advantages from the cell sheet transplantation not only on LV function but also on functional capacity and survival [149]. Moreover, the cell sheet prepared by hiPS-CMs has also been approved for clinical trials to treat heart disease patients [150, 151]. These results provide strong support for the practical application of cell sheets in treating “no-option” cardiac disease.

#### Clinical trials of cell sheets in other diseases

Cell sheets are reported in sealing air leaks, periodontal diseases, cutaneous ulcers, and knee osteoarthritis [33, 152]. Yamato et al*.* fabricated autologous dermal fibroblast sheets (DFS) and made their works as bio-artificial pleura and sealing air leaks in a patient with multiple bullae (Registration Number: UMIN000022554.). In 2018, Iwata et al*.* [152] evaluated the safety and effectiveness of cell sheets derived from autologous periodontal ligament (PDL) in clinical settings. They isolated PDL-derived cells from patients with periodontitis and transplanted the prepared PDL-derived cell sheets to the target area. During the mid-long-term follow-up period, no serious adverse events were found. Meanwhile, the periodontal defect was effectively healed, and the occlusal function was restored 6 months after transplantation. Hamano et al. [153] published the safety and effectiveness of cell-mixed sheets consisting of autologous fibroblast cells and peripheral blood mononuclear cells in cutaneous ulcers (Registration Number: UMIN000031645). The venous leg ulcers of two patients who had received the cell-mixed sheet transplantation decreased in size or healed.

## Conclusions and future outlook

CSE is a new technology that has shown promising potential in tissue regeneration. CSE uses special surfaces to form a dense cell sheet that can be detached under different stimulations such as temperature variation, magnetic force, controllable electrochemical change, and lightly applied to regulate cell adhesion and detachment. Multiple types of cell sheets can be prepared using various fabrication methods [138, 160–162], generating various kinds of 3D tissue constructs without using 3D scaffolds [154, 155]. In vitro vascularization using a vascular bed has also been successfully developed and enlarged the thickness and volume of cell-dense tissue without necrosis in the tissue center. Cell sheets can form structures mimicking tissues and organs, which further opens a way for personalization and precision medicine.

Products constructed using CSE technology retain intact cell–cell junctions and associated ECM. Cell therapy based on this technology can deliver a variety of therapeutic cells to the injured site, and the grafts can stably exist in the preset position without suturing, thus, avoiding additional damage to the body. Many preclinical studies have confirmed that cell sheet transplantation is superior in safety and efficiency to cell suspension injection. Organoids are defined as “ a 3D structure derived from either PSCs, neonatal tissue stem cells or adult stem cells/adult progenitors spontaneously self-organize into properly differentiated functional cell types and progenitors, resembling their in vivo counterpart and recapitulating at least some function of the organ” [156, 157]. Organ buds fabricated by cells self-assembly of co-cultured cells (MSCs is essential as they drive cells condensation) are also a sort of Organoids[158]. Organoids are sphere-like structures that are not easy to adhere to the surface of organs, and the accumulation of grafts is not easy for the supply of oxygen and nutrients, which leads to the rising risks of necrosis. Moreover, self-organized cell clusters of organoids fabrication lack scalability or reproducibility in size and cellular organization. These problems cause the status organoids to be used sparingly in regenerative medicine [159]. The organoids technology is a novel culture strategy that better maintains cells in a near-native state and has apparent advantages in vitro drug screening and precision medicine [160]. Novel technologies such as 3D bio-printing may help to construct organoids-based products with larger sizes and more similar structures, which may be more advantageous in cell therapy. CSE harvested sheet-like structures. And the basis of CSE is novel cell harvest technology, and the application of cell sheets concentrates upon cell delivery of regenerative medicine [160]. Even though the complex multi-layered cell sheets or multi-cells-derived cell sheets are more complex in the cell compositions or structure, they remain sheet-like, cylinder-like, or cuboid-like structures and achieve a relatively large contact area, which is conducive to achieving adhesion in the host and have advantages in regenerative medicine[161]. Also, the application of CSE in ocular surface reconstruction, retinal photoreceptor regeneration, periodontal tissue repair, esophageal mucosa healing, and epidermal skin regeneration has entered clinical trials. However, CSE and organoids technology have their own advantages and are currently used more in regenerative medicine and complex in vitro models. Evidence shows that they can also realize the other’ s superior applications. For example, a cell-sheet-based in vitro NASH model containing hepatocytes, which are similar to ballooned hepatocytes in human nonalcoholic steatohepatitis (NASH), was established [63]. Also, the combined application of CSE and organoids technology may be a great solution for complex in vitro models (CIVM) formation and regenerative medicine.

CSE also has several limitations: (1) the cost of cell sheet fabrication is still high, which may limit the popularization of this technic; (2) compared with other transplantation strategies, the preparation and harvest of cell sheets or thicker tissue are time-consuming. Yet, the storage technique of cell products, cryopreservation [162] of cell sheets, has also been improved, reducing the cost of regenerative medicine and promoting its use. In recent years, researchers have been exploring more widely applicable cell resources and more economical preparation methods, developing and updating surface materials, and designing rapid preparation methods to solve cost and time problems. iPSCs/hESCs derived cells have been reported as new cell resources [54], which has already significantly reduced the price of this technology; new surface materials have been prepared to support the rapid preparation and harvest of cell sheets, which may shorten the fabrication time [163, 164].

Meanwhile, the development of 3D printing, organoids technology, and other emerging technologies has provided new opportunities for the design and manufacture of patterned cell sheet culture surfaces and culture vessels adapted to the requirements of the preparation of complex structures or systems [165–167], which further suggests that CSE could be an alternative to injury repair and participate in constructing drug research models and drug screening platforms. Advances in CSE technology are expected to expand the number of target diseases in regenerative medicine and enable us to produce constructs for treating diseases that traditional therapies cannot cure. In the future, pre-vascularized 3D tissues with complex structures and micropattern cell sheets that can rebuild tissue polarity and simulate native structures may be used. With the mutual support of various interdisciplinary disciplines, the reconstruction and regeneration of larger organizations are expected to be realized in the future, providing refractory diseases with new medical resources.

## Data Availability

Not applicable.

## Abbreviations

CS: Cell sheet
CSE: Cell sheet engineering
ECM: Extracellular matrix
SCID: Severe combined immunodeficiency
MSCs: Mesenchymal stem cells
ADSCs: Adipose tissue-derived stromal cells
hESCs: Human embryonic stem cells
hiPSCs: Human induced pluripotent stem cells
NASH: Nonalcoholic steatohepatitis
HUVECs: Human umbilical vein endothelial cells
CMs: Cardiomyocytes
hiPS-CMs: Human iPSCs derived cardiomyocytes
OMECs: Oral mucosa cells
COMECs: Cultured oral mucosa cells
RPE: Retinal pigment epithelium
LSCD: Limbal stem cell deficiency
NGMA: N-isopropylacrylamide-co-glycidylmethacrylate
EHFSs: Engineered two-layer hepatocyte/fibroblast sheet
PHH: Primary human hepatocytes
HSC: Hepatic stellate cells
hDPSCs: Human dental pulp stem cells
hTDM: Human treated dentin matrix
PDLCs: Periodontal ligament cells
SMG: Submandibular gland
ASCs: Adipose-derived stromal cells
STSGs: Split-thickness skin grafts
ESD: Endoscopic submucosal dissection
TFBC: Tendon-fibrocartilage-bone composite
hAMSCs: Human amniotic mesenchymal stem cells
HGF: Hepatocyte growth factor
CKD: Chronic kidney disease
SJS: Stevens–Johnson Syndrome
AMD: Age-related macular degeneration
DCM: Dilated cardiomyopathy
LVAS: Left ventricular assist system
LV: Left ventricular
PDL: Periodontal ligament
CCL_4_: Carbon tetrachloride
hUC-MSCs: Human umbilical cord mesenchymal stem cells
MI: Myocardial infarction
DSPP: Dentin sialophosphoprotein
OCN: Osteocalcin
VEGFR1: Vascular endothelial growth factor receptor 1
SHEDs: Stem cells from human exfoliated deciduous teeth
PHCS: Pre-vascularized hMSC cell sheets
hFIX: Human coagulation factor IX
BOECs: Blood outgrowth endothelial cells
LVEF: Left ventricular ejection fraction
DFS: Dermal fibroblast sheets

## References

[1] Facklam AL, Volpatti LR, Anderson DG (2020). Biomaterials for personalized cell therapy. Adv Mater.

[2] Nguyen PK, Rhee JW, Wu JC (2016). Adult stem cell therapy and heart failure, 2000 to 2016: a systematic review. JAMA Cardiol.

[3] Shetty AK (2018). Emerging anti-aging strategies—scientific basis and efficacy. Aging Dis.

[4] Behfar A (2014). Cell therapy for cardiac repair—lessons from clinical trials. Nat Rev Cardiol.

[5] Liu E (2020). Use of CAR-transduced natural killer cells in CD19-positive lymphoid tumors. N Engl J Med.

[6] Mikkilineni L, Kochenderfer JN (2021). CAR T cell therapies for patients with multiple myeloma. Nat Rev Clin Oncol.

[7] Hu C (2019). Strategies to improve the efficiency of mesenchymal stem cell transplantation for reversal of liver fibrosis. J Cell Mol Med.

[8] Rebelatto CLK (2022). Safety and long-term improvement of mesenchymal stromal cell infusion in critically COVID-19 patients: a randomized clinical trial. Stem Cell Res Ther.

[9] Cannon JW (2018). Hemorrhagic shock. N Engl J Med.

[10] Shadman M (2022). Autologous transplant vs chimeric antigen receptor T-cell therapy for relapsed DLBCL in partial remission. Blood.

[11] Salhotra A (2020). Mechanisms of bone development and repair. Nat Rev Mol Cell Biol.

[12] Rothe R (2021). A modular, injectable, non-covalently assembled hydrogel system features widescale tunable degradability for controlled release and tissue integration. Biomaterials.

[13] Shao L (2020). Sacrificial microgel-laden bioink-enabled 3D bioprinting of mesoscale pore networks. Bio-Des Manuf.

[14] Vacanti CA, Vacanti JP (2000). The science of tissue engineering. Orthop Clin N Am.

[15] Skog M (2019). The effect of enzymatic digestion on cultured epithelial autografts. Cell Transplant.

[16] Miersch C, Stange K, Röntgen M (2018). Effects of trypsinization and of a combined trypsin, collagenase, and DNase digestion on liberation and in vitro function of satellite cells isolated from juvenile porcine muscles. In Vitro Cell Dev Biol Anim.

[17] Chen L, Deng C, Li J (2018). 3D printing of a lithium-calcium-silicate crystal bioscaffold with dual bioactivities for osteochondral interface reconstruction. Biomaterials.

[18] Kirby GTS (2018). Cell sheets in cell therapies. Cytotherapy.

[19] Yang J (2005). Cell sheet engineering: recreating tissues without biodegradable scaffolds. Biomaterials.

[20] Sekine W, Haraguchi Y (2011). Thickness limitation and cell viability of multi-layered cell sheets and overcoming the diffusion limit by a porous-membrane culture insert. J Biochips Tissue Chips.

[21] Wang L (2022). Bone marrow mesenchymal stem cell sheets with high expression of hBD3 and CTGF promote periodontal regeneration. Biomater Adv.

[22] Matsuo N (2023). Transplantation of hybrid adipose-derived stem cell sheet with autologous peritoneum: an in vivo feasibility study. Heliyon.

[23] Kaibuchi N (2022). Novel cell therapy using mesenchymal stromal cell sheets for medication-related osteonecrosis of the jaw. Front Bioeng Biotechnol.

[24] Kimura M (2023). Regeneration using adipose-derived stem cell sheets in a rabbit meniscal defect model improves tensile strength and load distribution function of the meniscus at 12 weeks. Arthroscopy.

[25] Yamaguchi S (2022). Highly feasible procedure for laparoscopic transplantation of cell sheets under pneumoperitoneum in porcine model. Surg Endosc.

[26] Riehle C, Bauersachs J (2019). Small animal models of heart failure. Cardiovasc Res.

[27] Zhang W, Yang W, Liu X (2014). Rapidly constructed scaffold-free embryonic stem cell sheets for ocular surface reconstruction. Scanning.

[28] Ohki T, Yamato M, Murakami D (2006). Treatment of oesophageal ulcerations using endoscopic transplantation of tissue-engineered autologous oral mucosal epithelial cell sheets in a canine model. Gut.

[29] Yao M (2022). Natural tissue-imprinted biointerface for the topographical education of a biomimetic cell sheet. Langmuir.

[30] Ebihara G, Sato M, Yamato M (2012). Cartilage repair in transplanted scaffold-free chondrocyte sheets using a minipig model. Biomaterials.

[31] Kawamura M, Miyagawa S, Fukushima S (2017). Enhanced therapeutic effects of human iPS cell derived-cardiomyocyte by combined cell-sheets with omental flap technique in porcine ischemic cardiomyopathy model. Sci Rep.

[32] Kobayashi S (2022). Allogeneic transplantation of epidermal cell sheets followed by endoscopic submucosal dissection to prevent severe esophageal stricture in a porcine model. Regen Ther.

[33] Sato M (2019). Combined surgery and chondrocyte cell-sheet transplantation improves clinical and structural outcomes in knee osteoarthritis. NPJ Regen Med.

[34] Fujita A (2019). Hypoxic-conditioned cardiosphere-derived cell sheet transplantation for chronic myocardial infarction. Eur J Cardiothorac Surg.

[35] Oliva J (2019). Vitrification and storage of oral mucosa epithelial cell sheets. J Tissue Eng Regen Med.

[36] Imafuku A (2019). Rat mesenchymal stromal cell sheets suppress renal fibrosis via microvascular protection. Stem Cells Transl Med.

[37] Roh JL (2017). Plasticity of oral mucosal cell sheets for accelerated and scarless skin wound healing. Oral Oncol.

[38] Robinson NB (2019). The current state of animal models in research: a review. Int J Surg.

[39] Saito T (2011). Reversal of diabetes by the creation of neo-islet tissues into a subcutaneous site using islet cell sheets. Transplantation.

[40] Nagamoto Y, Takayama K, Ohashi K (2016). Transplantation of a human iPSC-derived hepatocyte sheet increases survival in mice with acute liver failure. J Hepatol.

[41] Kuramoto G (2018). Endometrial regeneration using cell sheet transplantation techniques in rats facilitates successful fertilization and pregnancy. Fertil Steril.

[42] Iqbal MA (2019). Severe combined immunodeficiency pig as an emerging animal model for human diseases and regenerative medicines. BMB Rep.

[43] Song J (2013). Generation of RAG 1- and 2-deficient rabbits by embryo microinjection of TALENs. Cell Res.

[44] Hashikawa Y (2020). Generation of knockout rabbits with X-linked severe combined immunodeficiency (X-SCID) using CRISPR/Cas9. Sci Rep.

[45] Lee K (2014). Engraftment of human iPS cells and allogeneic porcine cells into pigs with inactivated RAG2 and accompanying severe combined immunodeficiency. Proc Natl Acad Sci USA.

[46] Kang JT (2016). Biallelic modification of IL2RG leads to severe combined immunodeficiency in pigs. Reprod Biol Endocrinol.

[47] Eizirik DL, Pasquali L, Cnop M (2020). Pancreatic beta-cells in type 1 and type 2 diabetes mellitus: different pathways to failure. Nat Rev Endocrinol.

[48] Hirabaru M, Kuroki T, Adachi T (2015). A method for performing islet transplantation using tissue-engineered sheets of islets and mesenchymal stem cells. Tissue Eng Part C Methods.

[49] Matsushima H, Kuroki T, Adachi T (2016). Human fibroblast sheet promotes human pancreatic islet survival and function in vitro. Cell Transplant.

[50] Lee YN (2021). Improvement of the therapeutic capacity of insulin-producing cells trans-differentiated from human liver cells using engineered cell sheet. Stem Cell Res Ther.

[51] Lee YN (2020). Evaluation of multi-layered pancreatic islets and adipose-derived stem cell sheets transplanted on various sites for diabetes treatment. Cells.

[52] Xu B (2019). Three-dimensional culture promotes the differentiation of human dental pulp mesenchymal stem cells into insulin-producing cells for improving the diabetes therapy. Front Pharmacol.

[53] Sui L (2018). β-Cell replacement in mice using human type 1 diabetes nuclear transfer embryonic stem cells. Diabetes.

[54] Du Y (2022). Human pluripotent stem-cell-derived islets ameliorate diabetes in non-human primates. Nat Med.

[55] Nilsson M, Fagman H (2017). Development of the thyroid gland. Development.

[56] Huang Y (2019). Fabrication of functional cell sheets with human thyrocytes from non-tumorous thyroid tissue. Tissue Eng Regen Med.

[57] Itaba N (2015). Human mesenchymal stem cell-engineered hepatic cell sheets accelerate liver regeneration in mice. Sci Rep.

[58] Ohashi K, Yokoyama T, Yamato M (2007). Engineering functional two- and three-dimensional liver systems in vivo using hepatic tissue sheets. Nat Med.

[59] Sakai Y, Yamanouchi K, Ohashi K (2015). Vascularized subcutaneous human liver tissue from engineered hepatocyte/fibroblast sheets in mice. Biomaterials.

[60] Kim K, Utoh R, Ohashi K, Kikuchi T, Okano T (2017). Fabrication of functional 3D hepatic tissues with polarized hepatocytes by stacking endothelial cell sheets in vitro. J Tissue Eng Regen Med.

[61] Ohashi K, Koyama F, Tatsumi K (2010). Functional life-long maintenance of engineered liver tissue in mice following transplantation under the kidney capsule. J Tissue Eng Regen Med.

[62] Baimakhanov Z, Yamanouchi K, Sakai Y (2016). Efficacy of multilayered hepatocyte sheet transplantation for radiation-induced liver damage and partial hepatectomy in a rat model. Cell Transplant.

[63] Hasui N (2022). In vitro ballooned hepatocytes can be produced by primary human hepatocytes and hepatic stellate cell sheets. Sci Rep.

[64] Sakaguchi K, Shimizu T, Okano T (2015). Construction of three-dimensional vascularized cardiac tissue with cell sheet engineering. J Control Release.

[65] von Bornstädt D (2018). Rapid self-assembly of bioengineered cardiovascular bypass grafts from scaffold-stabilized, tubular bilevel cell sheets. Circulation.

[66] Kim SJ, Jun I, Kim DW (2013). Rapid transfer of endothelial cell sheet using a thermosensitive hydrogel and its effect on therapeutic angiogenesis. Biomacromol.

[67] Shudo Y, Cohen JE, Macarthur JW (2013). Spatially oriented, temporally sequential smooth muscle cell-endothelial progenitor cell bi-level cell sheet neovascularizes ischemic myocardium. Circulation.

[68] Kashiyama N (2022). Adipose-derived stem cell sheet under an elastic patch improves cardiac function in rats after myocardial infarction. J Thorac Cardiovasc Surg.

[69] Sekiya S, Morikawa S, Ezaki T, Shimizu T (2018). Pathological process of prompt connection between host and donor tissue vasculature causing rapid perfusion of the engineered donor tissue after transplantation. Int J Mol Sci.

[70] Shevchenko EK, Dergilev KV, Tsokolaeva ZI (2019). Combination of mesenchymal stromal cells and cardiac stem cells in a multilayer cell construct promotes activation of notch signaling and initiation of endothelial differentiation. Bull Exp Biol Med.

[71] Gao S (2022). Preclinical study of human umbilical cord mesenchymal stem cell sheets for the recovery of ischemic heart tissue. Stem Cell Res Ther.

[72] Nishida K, Yamato M, Hayashida Y (2004). Functional bioengineered corneal epithelial sheet grafts from corneal stem cells expanded ex vivo on a temperature-responsive cell culture surface. Transplantation.

[73] Yoshinaga Y (2022). Long-term survival in non-human primates of stem cell-derived, MHC-unmatched corneal epithelial cell sheets. Stem Cell Rep.

[74] Ma DH (2021). Long-term survival of cultivated oral mucosal epithelial cells in human cornea: generating cell sheets using an animal product-free culture protocol. Stem Cell Res Ther.

[75] Iraha S, Tu HY, Yamasaki S (2018). Establishment of immunodeficient retinal degeneration model mice and functional maturation of human ESC-derived retinal sheets after transplantation. Stem Cell Rep.

[76] Kamao H, Mandai M, Okamoto S (2014). Characterization of human induced pluripotent stem cell-derived retinal pigment epithelium cell sheets aiming for clinical application. Stem Cell Rep.

[77] Gu S (2009). Differentiation of rabbit bone marrow mesenchymal stem cells into corneal epithelial cells in vivo and ex vivo. Mol Vis.

[78] Venugopal B, Shenoy SJ, Mohan S, Anil-Kumar PR, Kumary TV (2020). Bioengineered corneal epithelial cell sheet from mesenchymal stem cells—a functional alternative to limbal stem cells for ocular surface reconstruction. J Biomed Mater Res B Appl Biomater.

[79] Kamao H, Mandai M, Ohashi W (2017). Evaluation of the surgical device and procedure for extracellular matrix-scaffold-supported human iPSC-derived retinal pigment epithelium cell sheet transplantation. Investig Ophthalmol Vis Sci.

[80] Ashok K (2021). Characterization and evaluation of ascorbic acid-induced cell sheet formation in human periodontal ligament stem cells: an in vitro study. J Oral Biosci.

[81] Hu J, Cao Y, Xie Y (2016). Periodontal regeneration in swine after cell injection and cell sheet transplantation of human dental pulp stem cells following good manufacturing practice. Stem Cell Res Ther.

[82] Wei F, Qu C, Song T (2012). Vitamin C treatment promotes mesenchymal stem cell sheet formation and tissue regeneration by elevating telomerase activity. J Cell Physiol.

[83] Meng H, Hu L, Zhou Y (2020). A sandwich structure of human dental pulp stem cell sheet, treated dentin matrix, and matrigel for tooth root regeneration. Stem Cells Dev.

[84] Li R, Guo W, Yang B (2011). Human treated dentin matrix as a natural scaffold for complete human dentin tissue regeneration. Biomaterials.

[85] Raju R, Oshima M, Inoue M (2020). Three-dimensional periodontal tissue regeneration using a bone-ligament complex cell sheet. Sci Rep.

[86] Nam K, Kim K, Dean SM (2019). Using cell sheets to regenerate mouse submandibular glands. NPJ Regen Med.

[87] Lee DY, Kim H-B, Shim IK (2017). Treatment of chemically induced oral ulcer using adipose-derived mesenchymal stem cell sheet. J Oral Pathol Med.

[88] Lee J-M, Kim H-Y, Park J-S (2019). Developing palatal bone using human mesenchymal stem cell and stem cells from exfoliated deciduous teeth cell sheets. J Tissue Eng Regen Med.

[89] Na J, Song SY, Kim JD (2018). Protein-engineered large area adipose-derived stem cell sheets for wound healing. Sci Rep.

[90] Benchaprathanphorn K (2020). Preparation and characterization of human keratinocyte-fibroblast cell sheets constructed using PNIAM-co-AM grafted surfaces for burn wound healing. J Mater Sci Mater Med.

[91] Lee J, Shin D, Roh JL (2018). Use of a pre-vascularised oral mucosal cell sheet for promoting cutaneous burn wound healing. Theranostics.

[92] Lee J, Shin D, Roh JL (2019). Promotion of skin wound healing using prevascularized oral mucosal cell sheet. Head Neck.

[93] Martins JM, de Oliveira FD, Lima EO, Dullius D, de Oliveira Durli IC, Hiraiwa E, Serrano T, Teixeira GR, Sampaio PM, Collares MV (2019). Use of derived adipose stem cells to reduce complications of cutaneous scarring in smokers. An experimental model in rats. Acta Cirurgica Brasilra.

[94] Yuka Kato TI, Morikawa S, Yamato S, Okano T, Uchigata Y (2015). Allogeneic transplantation of an adipose-derived stem cell sheet combined with artificial skin accelerates wound healing in a rat wound model of type 2 diabetes and obesity. Diabetes.

[95] Zhang Y, Li D, Fang S (2019). Stimulatory effect of engineered three-layer adipose tissue-derived stem cells sheet in atelocollagen matrix on wound healing in a mouse model of radiation-induced skin injury. J Biomater Appl.

[96] Lee YJ, Baek SE, Lee S (2019). Wound-healing effect of adipose stem cell-derived extracellular matrix sheet on full-thickness skin defect rat model: histological and immunohistochemical study. Int Wound J.

[97] Zhang L, Xing Q, Qian Z (2016). Hypoxia created human mesenchymal stem cell sheet for prevascularized 3D tissue construction. Adv Healthc Mater.

[98] Chen L, Radke D, Qi S, Zhao F (2019). Protocols for full thickness skin wound repair using prevascularized human mesenchymal stem cell sheet. Methods Mol Biol.

[99] Chen L, Xing Q, Zhai Q (2017). Pre-vascularization enhances therapeutic effects of human mesenchymal stem cell sheets in full thickness skin wound repair. Theranostics.

[100] Aleksandrushkina NA, Danilova NV, Grigorieva OA (2019). Cell sheets of mesenchymal stromal cells effectively stimulate healing of deep soft tissue defects. Bull Exp Biol Med.

[101] Draganov PV (2019). AGA institute clinical practice update: endoscopic submucosal dissection in the United States. Clin Gastroenterol Hepatol.

[102] Perrod G, Rahmi G, Pidial L (2016). Cell sheet transplantation for esophageal stricture prevention after endoscopic submucosal dissection in a porcine model. PLoS ONE.

[103] Mervis JS, Phillips TJ (2019). Pressure ulcers: pathophysiology, epidemiology, risk factors, and presentation. J Am Acad Dermatol.

[104] Yu N (2021). Pressure injury: a non-negligible comorbidity for critical Covid-19 patients. J Plast Reconstr Aesthet Surg.

[105] Fryer S (2023). Continuous pressure monitoring of inpatient spinal cord injured patients: implications for pressure ulcer development. Spinal Cord.

[106] Bukowska J (2020). Safety and efficacy of human adipose-derived stromal/stem cell therapy in an immunocompetent murine pressure ulcer model. Stem Cells Dev.

[107] Xiao S (2019). Diabetic human adipose-derived stem cells accelerate pressure ulcer healing by inducing angiogenesis and neurogenesis. Stem Cells Dev.

[108] Badiavas EV, Falanga V (2003). Treatment of chronic wounds with bone marrow-derived cells. Arch Dermatol.

[109] Alexandrushkina N (2020). Cell sheets from adipose tissue MSC induce healing of pressure ulcer and prevent fibrosis via trigger effects on granulation tissue growth and vascularization. Int J Mol Sci.

[110] Yu J (2018). Cell sheet composed of adipose-derived stem cells demonstrates enhanced skin wound healing with reduced scar formation. Acta Biomater.

[111] Kim N (2020). Therapeutic effects of platelet derived growth factor overexpressed-mesenchymal stromal cells and sheets in canine skin wound healing model. Histol Histopathol.

[112] Maruya Y (2017). Autologous adipose-derived stem cell sheets enhance the strength of intestinal anastomosis. Regen Ther.

[113] Hara T, Soyama A, Adachi T (2020). Ameliorated healing of biliary anastomosis by autologous adipose-derived stem cell sheets. Regen Ther.

[114] Tanaka Takayuki KT, Tomohiko A (2013). Development of a novel rat model with pancreatic fistula and the prevention of this complication using tissue-engineered myoblast sheets. J Gastroenterol.

[115] Yoo JC, Ahn JH, Koh KH, Lim KS (2009). Rotator cuff integrity after arthroscopic repair for large tears with less-than-optimal footprint coverage. Arthrosc J Arthrosc Relat Surg.

[116] Yang G, Rothrauff BB, Tuan RS (2013). Tendon and ligament regeneration and repair: clinical relevance and developmental paradigm. Birth Defects Res C Embryo Today.

[117] Liu Q, Yu Y, Reisdorf RL (2019). Engineered tendon-fibrocartilage-bone composite and bone marrow-derived mesenchymal stem cell sheet augmentation promotes rotator cuff healing in a non-weight-bearing canine model. Biomaterials.

[118] You Q, Liu Z, Zhang J (2020). Human amniotic mesenchymal stem cell sheets encapsulating cartilage particles facilitate repair of rabbit osteochondral defects. Am J Sports Med.

[119] Silva AS (2020). Multi-layer pre-vascularized magnetic cell sheets for bone regeneration. Biomaterials.

[120] Yoon Y, Jung T, Afan Shahid M (2019). Frozen-thawed gelatin-induced osteogenic cell sheets of canine adipose-derived mesenchymal stromal cells improved fracture healing in canine model. J Vet Sci.

[121] Falkevall A, Mehlem A, Palombo I (2017). Reducing VEGF-B signaling ameliorates renal lipotoxicity and protects against diabetic kidney disease. Cell Metab.

[122] Toyohara T, Mae SI, Sueta SI (2015). Cell therapy using human induced pluripotent stem cell-derived renal progenitors ameliorates acute kidney injury in mice. Stem Cells Transl Med.

[123] Takemura S (2020). Transplantation of adipose-derived mesenchymal stem cell sheets directly into the kidney suppresses the progression of renal injury in a diabetic nephropathy rat model. J Diabetes Investig.

[124] Oka M (2019). Hepatocyte growth factor-secreting mesothelial cell sheets suppress progressive fibrosis in a rat model of CKD. J Am Soc Nephrol.

[125] Imafuku A, Oka M, Miyabe Y (2019). Rat mesenchymal stromal cell sheets suppress renal fibrosis via microvascular protection. Stem Cells Transl Med.

[126] Kawanishi K (2016). Peritoneal cell sheets composed of mesothelial cells and fibroblasts prevent intra-abdominal adhesion formation in a rat model. J Tissue Eng Regen Med.

[127] Mancuso ME, Mahlangu JN, Pipe SW (2021). The changing treatment landscape in haemophilia: from standard half-life clotting factor concentrates to gene editing. Lancet.

[128] Watanabe N, Ohashi K, Tatsumi K (2013). Genetically modified adipose tissue-derived stem/stromal cells, using simian immunodeficiency virus-based lentiviral vectors, in the treatment of hemophilia B. Hum Gene Ther.

[129] Tatsumi K, Sugimoto M, Lillicrap D (2013). A novel cell-sheet technology that achieves durable factor VIII delivery in a mouse model of hemophilia A. PLoS ONE.

[130] Takagi S, Shimizu T, Kuramoto G (2014). Reconstruction of functional endometrium-like tissue in vitro and in vivo using cell sheet engineering. Biochem Biophys Res Commun.

[131] Kuramoto G, Takagi S, Ishitani K (2015). Preventive effect of oral mucosal epithelial cell sheets on intrauterine adhesions. Hum Reprod.

[132] Kuramoto G (2022). Human mesenchymal stem cell sheets improve uterine incision repair in a rodent hysterotomy model. Am J Perinatol.

[133] Kuramoto G, Shimizu T, Takagi S (2018). Endometrial regeneration using cell sheet transplantation techniques in rats facilitates successful fertilization and pregnancy. Fertil Steril.

[134] Takahashi H, Okano T (2019). Thermally-triggered fabrication of cell sheets for tissue engineering and regenerative medicine. Adv Drug Deliv Rev.

[135] Novak N, Haberstok J, Bieber T, Allam JP (2008). The immune privilege of the oral mucosa. Trends Mol Med.

[136] Grimsby GM, Bradshaw K, Baker LA (2014). Autologous buccal mucosa graft augmentation for foreshortened vagina. Obstet Gynecol.

[137] Bauer HK, Flesch D, Walenta S (2019). Primary mucosal epithelial cell cultivation: a reliable and accelerated isolation. Tissue Eng Part C Methods.

[138] Nishida K (2004). Corneal reconstruction with tissue-engineered cell sheets composed of autologous oral mucosal epithelium. N Engl J Med.

[139] Kim YJ, Lee HJ, Ryu JS (2017). Prospective clinical trial of corneal reconstruction with biomaterial-free cultured oral mucosal epithelial cell sheets. Cornea.

[140] Ohki T, Yamato M, Ota M (2012). Prevention of esophageal stricture after endoscopic submucosal dissection using tissue-engineered cell sheets. Gastroenterology.

[141] Yamaguchi N, Isomoto H, Kobayashi S (2017). Oral epithelial cell sheets engraftment for esophageal strictures after endoscopic submucosal dissection of squamous cell carcinoma and airplane transportation. Sci Rep.

[142] Jonas E, Sjoqvist S, Elbe P (2016). Transplantation of tissue-engineered cell sheets for stricture prevention after endoscopic submucosal dissection of the oesophagus. United Eur Gastroenterol J.

[143] Burillon C, Huot L, Justin V (2012). Cultured autologous oral mucosal epithelial cell sheet (CAOMECS) transplantation for the treatment of corneal limbal epithelial stem cell deficiency. Investig Ophthalmol Vis Sci.

[144] Takagi S, Mandai M, Gocho K (2019). Evaluation of transplanted autologous induced pluripotent stem cell-derived retinal pigment epithelium in exudative age-related macular degeneration. Ophthalmol Retina.

[145] Schwartz SD, Regillo CD, Lam BL (2015). Human embryonic stem cell-derived retinal pigment epithelium in patients with age-related macular degeneration and Stargardt's macular dystrophy: follow-up of two open-label phase 1/2 studies. Lancet.

[146] Sawa Y, Miyagawa S, Sakaguchi T (2012). Tissue engineered myoblast sheets improved cardiac function sufficiently to discontinue LVAS in a patient with DCM: report of a case. Surg Today.

[147] Sawa Y, Yoshikawa Y, Toda K (2015). Safety and efficacy of autologous skeletal myoblast sheets (TCD-51073) for the treatment of severe chronic heart failure due to ischemic heart disease. Circ J.

[148] Miyagawa S, Domae K, Yoshikawa Y (2017). Phase I clinical trial of autologous stem cell-sheet transplantation therapy for treating cardiomyopathy. J Am Heart Assoc.

[149] Kainuma S (2021). Long-term outcomes of autologous skeletal myoblast cell-sheet transplantation for end-stage ischemic cardiomyopathy. Mol Ther.

[150] Cyranoski D (2018). Reprogrammed stem cells approved to mend hearts. Nature.

[151] Domae K (2021). Clinical outcomes of autologous stem cell-patch implantation for patients with heart failure with nonischemic dilated cardiomyopathy. J Am Heart Assoc.

[152] Iwata T, Yamato M, Washio K (2018). Periodontal regeneration with autologous periodontal ligament-derived cell sheets—a safety and efficacy study in ten patients. Regen Ther.

[153] Mizoguchi T (2021). A pilot study using cell-mixed sheets of autologous fibroblast cells and peripheral blood mononuclear cells to treat refractory cutaneous ulcers. Am J Transl Res.

[154] Williams NP, Rhodehamel M, Yan C (2020). Engineering anisotropic 3D tubular tissues with flexible thermoresponsive nanofabricated substrates. Biomaterials.

[155] Jiao A, Moerk CT, Penland N (2018). Regulation of skeletal myotube formation and alignment by nanotopographically controlled cell-secreted extracellular matrix. J Biomed Mater Res A.

[156] Huch M, Koo BK (2015). Modeling mouse and human development using organoid cultures. Development.

[157] Tang XY (2022). Human organoids in basic research and clinical applications. Signal Transduct Target Ther.

[158] Takebe T (2013). Vascularized and functional human liver from an iPSC-derived organ bud transplant. Nature.

[159] Rossen NS (2020). Injectable therapeutic organoids using sacrificial hydrogels. iScience.

[160] LeSavage BL (2022). Next-generation cancer organoids. Nat Mater.

[161] Asakawa N (2010). Pre-vascularization of in vitro three-dimensional tissues created by cell sheet engineering. Biomaterials.

[162] Ike S (2022). Cryopreserved allogenic fibroblast sheets: development of a promising treatment for refractory skin ulcers. Am J Transl Res.

[163] Jiang Z (2021). Recent advances in light-induced cell sheet technology. Acta Biomater.

[164] Koo MA (2018). Exogenous ROS-induced cell sheet transfer based on hematoporphyrin-polyketone film via a one-step process. Biomaterials.

[165] Su W (2022). A microfluidic cell chip for virus isolation via rapid screening for permissive cells. Virol Sin.

[166] Li Q (2021). 3D printed silk-gelatin hydrogel scaffold with different porous structure and cell seeding strategy for cartilage regeneration. Bioact Mater.

[167] Chen X (2022). Harnessing 4D printing bioscaffolds for advanced orthopedics. Small.

